# New Advances in Nano-Drug Delivery Systems: *Helicobacter pylori* and Gastric Cancer

**DOI:** 10.3389/fonc.2022.834934

**Published:** 2022-05-10

**Authors:** Xiang Zhu, Tingting Su, Shouhua Wang, Huiqing Zhou, Weibin Shi

**Affiliations:** ^1^ Department of General Surgery, Xinhua Hospital, Shanghai Jiao Tong University School of Medicine, Shanghai, China; ^2^ Department of Gastroenterology, Xinhua Hospital, Shanghai Jiao Tong University School of Medicine, Shanghai, China

**Keywords:** nanoparticles, nanomaterials, nano-drug delivery systems, gastric cancer, *Helicobacter pylori*

## Abstract

With the development of materials science and biomedicine, the application of nanomaterials in the medical field is further promoted. In the process of the diagnosis and treatment of diseases, a variety of drugs need to be used. It is an ideal state to make these drugs arrive at a specific location at a specific time and release at a specific speed, which can improve the bioavailability of drugs and reduce the adverse effects of drugs on normal tissues. Traditional drug delivery methods such as tablets, capsules, syrups, and ointments have certain limitations. The emergence of a new nano-drug delivery system further improves the accuracy of drug delivery and the efficacy of drugs. It is well known that the development of the cancer of the stomach is the most serious consequence for the infection of *Helicobacter pylori*. For the patients who are suffering from gastric cancer, the treatments are mainly surgery, chemotherapy, targeted and immune therapy, and other comprehensive treatments. Although great progress has been made, the diagnosis and prognosis of gastric cancer are still poor with patients usually diagnosed with cancer at an advanced stage. Current treatments are of limited benefits for patients, resulting in a poor 5-year survival rate. Nanomaterials may play a critical role in early diagnosis. A nano-drug delivery system can significantly improve the chemotherapy, targeted therapy, and immunotherapy of advanced gastric cancer, reduce the side effects of the original treatment plan and provide patients with better benefits. It is a promising treatment for gastric cancer. This article introduces the application of nanomaterials in the diagnosis and treatment of *H. pylori* and gastric cancer.

## Introduction

Gastric cancer is the fifth most common cancer and fourth most deadly cancer in the world, causing more than 1 million new cases and about 769,000 deaths worldwide in 2020 (1). At present, the etiology of gastric cancer is not very clear, but it is related to regional, dietary, and genetic factors, *Helicobacter pylori* infection, and chronic diseases (2).

Gastric cancer can be divided into two types according to the site of carcinoma occurrence, cardia cancer, and non-cardia cancer. They differ in risk factors, carcinogenesis, and epidemiology. Patients with chronic *H. pylori* infection will develop non-cardiac cancer with serious consequences (1). *H. pylori* can cause gastric mucosa inflammation and injury through many ways and has a carcinogenic effect. Around half of the world’s population is infected with *H. pylori*, a high rate of infection (3). At present, great attention has been paid to the early control of *H. pylori* infection in order to achieve the prevention and treatment of gastric cancer. In addition, finding molecular markers that can predict early gastric cancer can achieve early detection, diagnosis, and treatment of gastric cancer patients. Because of the low sensitivity and specificity of current gastric cancer serum tumor markers, molecular markers such as protein, cell, DNA, and RNA have good clinical application prospects according to current research findings (**Table 1**) .

**Table 1:** A brief summary of potential biomarkers for early diagnosis of gastric cancer.

Level	Biomarkers	Reference
DNA	p16、RASSF10、RASSF1A、RPRM、RUNX3	(4–7)
miRNA	miR-196a、miR-196b、miR-19、miR-21、miR-17、miR-222 miR-146、miR-375、miR-101-3p	(8–11)
cirRNA	hsa-circ-0001017 hsa-circ-0061276 hsa-circ-0000026 has-circ-0000181	(12–14)
lncRNA	CUDR(19p13.12)、HOTAIR(12q13.13) BLACAT1(1q32.1) CCAT1(8q24.21) GAPLINC(18)	(15–17)
Protein	gastrokine 1(GKN1) 、flotillin 1、ADAM12 、TFF1	(18–22)
Cell	CTCs、cf DNA、TRIM3、miR-1246 lnc UEGC1	(23–27)

The treatment strategy of gastric cancer is comprehensive treatment with surgery as the main way. At present, chemotherapy, immunotherapy, and targeted therapy have become the focus of comprehensive treatment of gastric cancer. Traditional chemotherapeutic drugs are highly toxic and cause serious damage to normal cells due to their poor ability to recognize tumor cells. Drug resistance can also develop after several doses of the drug (28, 29). Immunotherapy has attracted worldwide attention due to its anticancer effect but has not been shown to have the same effect on all cancer patients (30).Although it has developed rapidly in recent years, great challenges have also been encountered in some specific situations, such as the drug resistance of immune checkpoint inhibitors and weak immunogenicity of therapeutic vaccines (31, 32).

The development of nanomaterial technology and biotechnology has promoted the innovation of nanomedicine. The nano-delivery system could avoid the poor selectivity of cancer therapies currently in use, which can damage healthy cells, so that drugs can reliably reach tumor tissues and reduce side effects caused by off-target in clinical application. This is due to the advantages of nanomaterials such as small volume and large surface area (33), higher permeability and retention; they can show greater specificity and can be combined with a variety of biological materials to reduce their toxicity and improve their biocompatibility. It could be used in nano- delivery systems, cancer detection, imaging, and drug-controlled release to targeted drugs (34, 35). It can effectively retain and control drug release and improve the efficiency of cancer treatment (36). The electrical, mechanical, optical, magnetic, and biological properties of nanomaterials are being used in the diagnosis and treatment of cancer (37). A variety of nanomaterials with good anticancer potential and improved diagnostic levels have been developed.

The application of nanomaterials in the medical field is in line with the promotion of personalized precision therapy. A nano-drug delivery system’s advantages will also get more application in clinics. Therefore, this paper briefly summarizes the progress of the application of the nanomaterials and deliver the medication system in *H. pylori* and gastric cancer, providing a reference for individual therapy to patients.

## *H. pylori* and Nanomaterials

*H. pylori* is a spiral, Gram-negative microaerobe. The infection rate of *H. pylori* is more than 50% worldwide. Its means of transmission is not very clear; the most likely way between people is the mouth–mouth and dung–mouth travels (38, 39). The cause of the disease is caused by many factors, such as bacterial flagella, adhesion, urease, protease, vacuolar toxin, endotoxin, and other synergistic effects. *H. pylori* can survive in the strong acid environment in the stomach because the urease it produces breaks down urea into ammonia and carbon dioxide, and the gastric acid is neutralized by ammonia, thus enabling it to survive in the stomach with high acidity (40).

Nanomaterials could play a crucial role in the early detection and treatment of *H. pylori*. For example, an immune sensor for *H. pylori* was prepared for the early detection of cytotoxin-related gene antibodies (41). Jain U has developed super-sensitive electrochemical sensors for the early noninvasive detection of *H. pylori* in clinical samples (42). Nanomaterials can also be used to prepare nano-drug delivery systems and develop effective nanotherapy for *H. pylori* infection.

### Relationship Between *H. pylori* Infection and Gastric Cancer

*H. pylori* was first discovered in patients with gastritis in 1982 by J. Robin Warren and Barry Marshall (43), in which an infection with this type of bacteria is one of the main reasons for various gastroduodenal diseases (44), including gastritis, peptic ulcer disease, and gastric cancer. This bacterium secretes versatile bacterial colonization factors and virulence factors, leading to gastric epithelial cell damage. After infection, chronic gastritis is first caused, which further results in gastric ulcer and atrophy, and gastric cancer is developed in severe cases (45). Unless receiving the specific antibacterial treatment, patients will carry these bacteria for life. The World Health Organization classified *H. pylori* as a class 1 carcinogen in 1994 (46). So, experts think that the early detection of *H. pylori* infection and timely and effective use of antibiotics to kill *H. pylori* have great significance to prevent stomach carcinoma. The timely diagnosis and cure of *H. pylori* have attracted people’s attention.

### *H. pylori* Pathogenicity in Gastric Carcinogenesis

The pathogenic mechanism of *H. pylori* is very complicated, and the damage mechanism of *H. pylori* to the gastric mucosa and human body has not been completely understood. At present, the factors involved in the pathogenesis of *H. pylori* are classified into colonization factors and virulence factors. which damage gastric epithelial cells through outer proteins such as BabA, SabA, and many variants of outer membrane proteins (**Table 2**) (45, 47). When *H. pylori* attaches to stomach cells, it forms microcolonies on the surface of the stomach for nutrients to ensure that its initial colonization is successful (48). *H. pylori* expresses virulent proteins such as CagA and VacA that control the host’s immune system to escape immune detection (49). Studies reveal that in the presence of VacA, CagA accumulates in dysfunctional autophagosomes, providing a possible explanation for the synergistical work of VacA and CagA (50).

**Table 2:** The factors involved in the pathogenesis of *H. pylori*.

Colonization Factors	Virulence Factors
Blood group antigen-binding adhesin (BabA)	Cytotoxin-associated gene A (*Cag*A)
Sialic acid-binding adhesin (SabA)	Vacuolating cyto-toxin A(VacA)
Outer inflammatory protein (OipA)	γ -Glutamyl transpeptidase (gGT)
*H. pylori* outer membrane protein Q (HopQ)	High-temperature requirement protein A (HtrA)
	Urease
	outer membrane vesicles (OMV)

### Nanomaterials and Detection of *H. pylori*

The *Cag*A gene encodes immune dominant Cag protein, which causes gastric injury. Karakus et al. developed an immunochromatographic strip (ICTS) for detecting CagA antibodies in the sera of infected patients. Compared with PCR and commercial enzyme linked immunosorbent assay (ELISA), the sensitivity and specificity of ICTS were 95% and 100%, indicating that ICTS can be applied to rapidly detect patients with CagA-positive *H. pylori* infection. Without the need for an invasive method for testing (41), Gupta et al. promoted a novel *H. pylori* immunosensor to detect the CagA antibody, which is based on platinum nanoparticles (NPs). The new immune sensor has good accuracy, precision, reliability, and storage stability (51).

Jain et al. built an unlabeled electrochemical immunosensor for detecting *H. pylori* by a covalently immobilized antibody (CagA) on a nanomaterial-modified gold electrode. The results of *H. pylori* determination in human feces showed good accuracy. The support of nanometer technical for the development of electrochemical sensors with super sensitive and early detection of non-invasive clinical samples of *H. pylori* laid a solid foundation (42).

The BabA immunosensor was based on nanohybridization, which was synthesized by the electrodeposition of palladium NPs on a gold electrode. The stable BabA antigen–antibody complex, with its high sensitivity and non-invasive detection method, will have broad application prospects in the early detection of *H. pylori* (52).

### Nano-Drug Delivery System and *H. pylori* Therapy

The pathogenic potential of *H. pylori* has been confirmed in many laboratories since its first discovery, and the World Health Organization classified *H. pylori* as a class 1 carcinogen (46).

There was a parallel relationship between *H. pylori* infection and gastric cancer mortality. Therefore, the timely detection and eradication of *H. pylori* treatment have aroused people’s attention. It also has a preventive effect on the occurrence of gastric cancer. Due to the development of *H. pylori* resistance, quadruple therapy was recommended as a first-line treatment, which has a really higher eradication rate (bismuth plus PPI plus two antibiotics) (53).

Nanocarriers could protect antibiotics in harsh gastric environments. Amoxicillin is an antibiotic commonly used to eradicate *H. pylori*. Because it is easily degraded by stomach acid, it needs to be taken in large doses and used in combination with other drugs. Treatments sometimes fail because of side effects. Yang et al. built a nano-drug carrier that is composed of chitosan/poly(acrylic acid) particles, superparamagnetic iron oxide NPs, and amoxicillin (SPIO/AMO@PAA/CHI). It avoids the failure of treatment because of the easy degradation of antibiotics and the side effects caused by high dose administration. It can improve the stranded time of drugs in the stomach (54).

Biofilm formation is thought to be one of the causes of treatment failure. Gopalakrishnan et al. clarify that N-acyl homoserine lactonase-stabilized silver NPs inhibit quorum sensing (QS) by degradation, thereby reducing biofilm formation and improving the effect treatment. (55). Niaz prepared a nanodelivery system that is composed of a protein/polysaccharide core-shell crown. This compound can improve the stability and bioaccessibility of ϵ-polylysine (ϵ-PL) in the treatment of *H. pylori*. Therefore, this nanosystem can raise the efficacy of antibacterial agents and develop effective nanotherapies for gastric infection (56).

The studies of Jawed et al. indicated that curcumin has many biological functions with low bio-availability, which limits their uses as therapeutic for *H. pylori*. When it is encapsulated into a biocompatible copolymer, the PLGA NP might be used as potential therapeutics against *H. pylori* (57). Nanocarriers show therapeutic potential, owing to their mucosal penetrating capacities and modifiable properties (58).

As a matter of fact, the first step in the development of *H. pylori* pathogenicity is the adhesion to the gastric epithelium (59). It is very critical to design NPs with adhesion ability to mucosa for the treatment of *H. pylori*. For this reason, Arif et al. designed cysteine-conjugated chitosan (CysCS)/PMLA-targeting NPs that encapsulate amoxicillin. It can delay antibiotics release in gastric acid and can be targeted to mucosa. These results indicate that the multifunctional amoxicillin-loaded NPs have great potential for the effective treatment of *H. pylori* infection (60).

## Gastric Cancer and Nanomaterials

Gastric cancer is treated in different ways according to different stages of development. In the early stages of gastric cancer, the lesions are confined to the mucosa and submucosa, regardless of the size of their range and whether there is lymph node metastasis. Endoscopic submucosal dissection (ESD) and endoscopic mucosal resection (EMR) are minimally invasive treatments and can be used for the treatment of early gastric cancer (61). The main treatment methods for advanced gastric cancer, surgical treatment, radiotherapy and chemotherapy, immunotherapy, and targeted therapy, have gradually risen in status and become hot research in recent years (62). Nanomaterials, small chemicals with novel properties in at least one dimension on the scale of 1–100 nm, have also received great attention from researchers in recent years as a tool for cancer treatment. The application of nanotechnology in medicine has been greatly developed, and it also plays a unique role in the early diagnosis and comprehensive treatment of gastric cancer. There is room for improvement in diagnosis and treatment, and these advances hold great potential for the development of nanomaterials for cancer treatment. A number of nanomaterials are currently used for cancer treatment.

### Early Diagnosis of Gastric Cancer and Nanomaterials

Most gastric cancer patients have no typical clinical symptoms in the early stage, and the diagnosis is already advanced, which makes it difficult to completely remove the tumor. Early diagnosis and accurate personalized intervention are important questions to improve the prognosis of gastric cancer. Therefore, it is difficult to find effective and specific markers for the early diagnosis and treatment of gastric cancer. Magnetic nanomaterials have their own advantages in this aspect.

The relationship between SNP and the DNA specificity of some genes in gastric cancer treatment pathway was analyzed by using a magnetic nanomaterial molecular carrier. The *RAS-BRAF* gene was introduced on the surface of magnetic NPs, which is used to isolate and detect protein and pathogens. The results show that the system has good detection sensitivity and separation selectivity. Studies suggest that the *PLCE1* gene may be a susceptibility gene of tumor cells and is closely related to disease prognosis (63).

The dysregulation of microRNA concentration has been considered as a marker of disease. Decreased Mir-17 concentration has been found in gastric cancer. Therefore, rapid, specific, and simple microRNA quantification techniques are becoming key factors for early diagnosis and treatment. Miti et al. reported an immobilized gold NP for the detection of Mir-17. The process is fast, and the detection range is effective. This technique may be a promising alternative to traditional laboratory techniques for detection and quantification (64).

Zhuang et al. have developed biosensor CPs/AUNP-AUE, combined with a DNA capture probe to detect the Mir-100 content in the serum of GC patients. The biosensor detection of Mir-100 is highly specific and can be used as a reliable strategy for clinical gastric cancer detection (65).

### Nanomaterials and Chemotherapy

The common chemotherapeutic drugs for gastric cancer include fluorouracil (5-FU, S-1, and capecitabine), platinum (cisplatin and oxaliplatin), taxane (paclitaxel, docetaxel), topoisomerase inhibitors (irinotecan), and anthracene (epirubicin) (61)

At present, most of the first-generation nanochemotherapy reagents on the market use non-targeted delivery systems, which lack selectivity to tumor tissues and are distributed to the whole body after administration, with low bioavailability, limited efficacy, great damage to normal cells, and obvious side effects. An intelligent responsive drug delivery system is developed by using stimulus-responsive materials, which responds to changes in the internal environment of the lesion and carries out self-feedback to realize on-demand drug release, improve efficacy, and reduce side effects. Commonly used stimulus nano-drug delivery systems include acid response, reduction response, temperature response, light response, and enzyme response. After receiving the corresponding stimulus, the conformation of the drug changes, the separation between the drug and the carrier occurs, and the drug can achieve controlled release and play a role in the body.

Chemotherapy drug resistance is a difficult clinical challenge. Miao et al. developed zinc oxide nanoparticles (ZnO-NPs), which have been proven to be a promising anticancer drug. It can inhibit the proliferation, migration, and invasion of gastric cancer cells and induce apoptosis. ZnO-NP is a kind of potential drug candidate for gastric cancer therapy by inhibiting autophagy to reduce chemotherapy resistance (29).

Signal transducers and transcriptional activator 3 (STAT3) are key transcription factors that are overactivated in gastric cancer and play a crucial role in the induction of chemotherapy resistance. Zheng et al. developed polylactic acid-hydroxy acetic acid (PLGA) NPs for simultaneous codelivery of doxorubicin (DOX) and nifuratel. DNNPs have been shown to induce mitochondria-dependent apoptosis, inhibit STAT3 phosphorylation, and enhance the anticancer effect. It can inhibit the STAT3 pathway and amplify apoptosis (66).

Azimee et al. utilized the autophagy potential of TiO (2) NPs to improve the chemotherapy effect of 5-fluorouracil (5-FU) on human AGS gastric cells. In *in vitro* gastric cancer models, they confirmed the beneficial effect of TiO (2) NPs combined with chemotherapy, which lay a foundation for the development of a possible solution to cancer chemotherapy resistance. It was promising to improve the efficacy of current cancer treatment strategies (67).

Paclitaxel (Ptx) is one of the main chemotherapy drugs for advanced gastric cancer. Its clinical application is limited due to poor solubility. Yu et al. designed Ptx-NPs with more effective antitumor drug and fewer side effects, which are composed of methoxy poly (ethylene glycol) and poly (epsilon-caprolactone) NPs. The cytotoxicity of the treated group by Ptx-NPs was superior to that of the same dose of free Ptx. The Ptx-NPs group had more autophagy, which enhanced the anticancer effect. (68). Deng et al. developed a novel Se@Albumin composite NP. In mouse models, it can improve cisplatin-induced intestinal mucositis and will provide new information for clinical treatment (69).

Poor hydrophobicity of docetaxel and poor solubility of the PI3K/AKT signaling pathway inhibitor LY294002 limit their clinical application. Cai et al. developed polylactic acid/glycolic acid (PLGA) NPs with TXT and LY294002. *In vitro* and *in vivo* model experiments showed that PLGA(TXT+LY294002) showed controlled release, decreased proliferation ability, and increased apoptosis rate, showing tumor-targeting properties, non-toxic to main organs, controlled release, and targeted tumor, which provide a new idea for the targeted therapy of gastric cancer (70).

Gastric cancer stem cells (CSCs) have the characteristics of chemotherapy drug resistance and uncertain proliferation. Yao et al. developed novel CSCs targeting glioma-associated oncogene homolog 1 (Gli1) small interfering RNA (siRNA) NPs, which were shown to significantly inhibit the malignant features of CSC and specifically eliminated gastric CSCs, indicating that these novel delivery systems provide a promising therapeutic approach for gastric cancer treatment (71).

### Nanomaterials and Targeted Therapy

Molecular targeted therapy is an important treatment for advanced gastric cancer. Its target of the epidermal growth factor receptor (EGFR) and vascular endothelial growth factor receptor (VEGF) can prolong the overall survival of advanced gastric cancer. Although targeted therapy has made great progress in gastric cancer, only a few targeted drugs have been approved for clinical use, such as trastuzumab (anti-HER2 drugs) and apatinib (antiangiogenic pathway drugs) (72).

Abnormal overexpression of HER2 protein is associated with gastric cancer and is one of the factors contributing to poor prognosis of gastric cancer. Therefore, HER2 is a therapeutic target for gastric cancer (73).

Trastuzumab (Tmab) is a humanized monoclonal antibody that selectively targets the human epidermal growth factor receptor 2 (HER2), and while Tmab has shown improvement in patient prognosis, the acquired resistance to this drug remains an issue. Kanaya et al. developed gold NPs bound to Tmab by taking advantage of their *in vivo* stability and ease of surface modification and demonstrated their efficacy in treating HER2-positive, Tmab-resistant gastric cancer cell lines (74). Kubota et al. developed HER2-targeted AuNPs (Tmab-AuNPs) and showed that they have an effective antitumor effect against Tmab-resistant cell lines. In addition, when HER2 is overexpressed artificially, Tmab-AuNPs are effective against HER2-negative gastric cancer cell lines (75). Kubota et al. designed HER2-targeted AuNP (T-AUNP). Combined with Tmab, it is a promising approach to overcome Tmab resistance in gastric cancer (76). Zhang et al. developed a gold nanoshell drug carrier targeting HER-2 and immune adjuvant CPG sequence for the delivery and selective photothermal release of targeted genes in gastric cancer cells. This drug delivery system has better gene transduction ability and combination therapy effect, which is expected to become a translational therapy for gastric cancer (77).

Angiogenesis plays a critical role in tumor genesis, growth, and metastasis. The expression of vascular endothelial growth factor (VEGF) is high in most solid tumors, and many VEGF inhibitors have been used in clinics (78). The strong tyrosine kinase activity of VEGFR2 makes targeting VEGFR2 therapy an important strategy for antitumor therapy (79).

Apatinib, a small-molecule tyrosine kinase inhibitor targeting VEGFR2, is approved for the treatment of gastric cancer (80). Apatinib can selectively target VEGFR2 and bind to its intracellular binding site of ATP, blocking the corresponding signaling pathway, thereby inhibiting tumor angiogenesis. Long et al. developed nanocomplex LP-R/C@AC(LP: PH-responsive liposome R/C: mixed membrane A: apatinib C: cinobufagin), which killed tumor cells *in vitro* and also significantly inhibited tumor invasion and metastasis. In addition, it showed stronger antitumor activity in a mouse model of gastric cancer compared to a single agent. Emerging nanotechnology offers an alternative approach to building novel drug delivery systems with high targeting capability and solubility (81). In the Zhang et al. study, PLGA-based NPs coloaded Sal with apatinib (Apa), The surface of the NPs was modified with tumor-homing peptide (iVR1 peptides). Studies have shown that Sal can improve the chemical sensitivity of gastric cancer to Apa, and this compound can achieve efficient tumor-targeted delivery (82)

### Nanomaterials and Immunotherapy

The emergence of immunotherapy has changed the mode of tumor treatment (83, 84), which activates the body’s own immune system to kill tumor cells. Immunotherapy is regarded as a treatment or cure for certain types of cancer with promising strategies. Immunotherapy has become a research focus of tumor therapy in recent years, and a series of progress has been made, but the proportion of patients who respond to immunotherapy is still deficient. The cause of this phenomenon is the production of immune tolerance. On the one hand, because of the low immunogenicity of tumor cells, less specific antigen on the surface; on the other hand, there are less immune cells within the tumor, due to the immunosuppressive microenvironment in the tumor (85), In view of the above two reasons, there are two steps to improve the tolerance of tumor immunotherapy. The first step is to enhance the immunogenicity of tumor tissue and improve the response rate. The second step is to regulate the tumor-immunosuppressive microenvironment and overcome the immune tolerance. The nano-drug delivery system plays a crucial role in improving immune tolerance. which can reverse immunosuppression and activate tumor immune response to play a role, increasing the traditional immunotherapy treatment effect, reducing the immune treatment of adverse reactions (86, 87). The role of several nanomaterials in immunotherapy is described below.

As one of the most promising immunotherapies, immune checkpoint inhibitors have attracted attention. Immune checkpoint therapy has made a remarkable clinical development in the cure of cancer (88). As the most thoroughly studied class of immunotherapies to date, immune checkpoint molecules, cytotoxic T lymphocyte antigen 4 (CTLA-4), and programmed cell death protein 1 (PD-1) have received special attention. Pd-1 and CTLA-4, which has already become the approved target of cancer therapy (89, 90).

Pd-l1 is a symbol of poor prognosis in gastric cancer (91). Luo et al. developed siRNA delivery systems for PD-L1 knockdown to achieve effective therapy and diagnosis in gastric cancer (92).

NPs play a critical role in enhancing immune regulation by integrating various molecules. NPs can manipulate immune cells by promoting targeted delivery to release drugs, antigens, and adjuvants at their intended target sites and escaping the pathological physiological disorders at the same time (84, 93, 94); therefore, drugs can be mediated by NP-potent drug therapeutic potential to expand treatment drugs.

### Applications of Nanomaterials in Other Aspects of Gastric Cancer

Photodynamic therapy is a novel approach to treat tumors. After photosensitive drugs are delivered to tumor tissues, the photosensitive agents are activated and reactive oxygen species are generated to produce toxic effects on tumor cells after local irradiation with appropriate wavelength light (95, 96). This treatment has fewer side effects and can achieve precise and effective treatment. It is considered as a promising approach for the treatment of cancer (97). Yang et al. used the cell membrane (CM) from SGC7901 cells to decorate silica nanoparticles (SLNs) and load the photodynamic agent chloride ion e6 (Ce6) to construct CM/SLN/Ce6 suitable for gastric cancer tumor targeting PDT. SGC7901 cells were specifically targeted *in vitro* and *in vivo*. It has a better anticancer effect. This may be an effective tumor-targeted photodynamic therapy for gastric carcinoma platforms (97).

Ultrasound-focused chemotherapy is a non-invasive treatment with excellent penetrating performance, which is used to treat deep tumors. However, it will inevitably cause harm to normal tissues around the lesions. Low-intensity focused ultrasound (LIFU) was used to overcome this problem, achieving precisely controlled imaging and treatment. Liu et al. developed a simple, versatile nanoplatform (DPP-R) that responds to LIFU and simultaneously provides targeted drug delivery. Its targeting ability, imaging function, and antitumor effect have been approved *in vitro* and *in vivo*. DPP-R combined with LIFU is a new treatment method for gastric cancer (98).

The carbon nanolayer tracer method can improve the lymph node black staining rate and lymph node detection rate and improve the accuracy of gastric cancer staging (99). It is safe and feasible to apply neoadjuvant chemotherapy in D2 radical gastrectomy for gastric cancer. It can increase the number of lymph nodes detected, which is conducive to the evaluation of chemotherapy effects and prognosis of patients (100).

## Discussion

The further application of nanomaterial synthesis technology in the biomedical field is due to advances in materials science, medicine and biomedicine. We have applied the properties of nanomaterials themselves to the biomedical field, showing unique advantages in the early diagnosis of *H. pylori* and gastric cancer. It can also be used to improve the deficiency of original treatment, reduce the side effects of drugs, and provide new methods for the prevention, early diagnosis, and treatment of gastric cancer. The application of these nanomaterials is in line with the requirements of individualized precision therapy advocated today. Nanodelivery systems provide new ideas and new approaches for individual therapy.

However, the transformation of nanomaterials into clinical applications comes with a number of challenges, including stability, gastrointestinal degradation, and immune response. Some nanomaterials are not biodegradable in the human body and their impact on human health is unclear. For example, metal NPs will remain in the body after using them for a long time, and they may increase the cytotoxicity. There are no uniform standards for nano-drug delivery systems. Nanomaterials must be biodegradable and biocompatible to obtain better clinical application value. A simple NP design and quality control mechanisms are essential for the clinical transformation of nanomedicine.

Although currently, there are limitations in their use, many related studies are being carried out, and nanomedicine has shown great efficacy improvement in preclinical studies. It is believed that more nanomaterials will be designed and approved in clinical trials soon. In the future, nanomaterials will be more widely used in the biomedical field and provide more therapeutic effects for patients in need by seriously addressing the issues related to nanomaterial design and clinical trial design.

## Author Contributions

All authors listed have made a substantial, direct, and intellectual contribution to the work and approved it for publication.

## Funding

This research was funded by Science and Technology Commission of shanghai municipality, (grant number 19140903602). The APC was funded by Science and Technology Commission of shanghai municipality.

## Conflict of Interest

The authors declare that the research was conducted in the absence of any commercial or financial relationships that could be construed as a potential conflict of interest.

## Publisher’s Note

All claims expressed in this article are solely those of the authors and do not necessarily represent those of their affiliated organizations, or those of the publisher, the editors and the reviewers. Any product that may be evaluated in this article, or claim that may be made by its manufacturer, is not guaranteed or endorsed by the publisher.
